# Early peripheral blood MCEMP1 and HLA-DRA expression predicts COVID-19 prognosis

**DOI:** 10.1016/j.ebiom.2023.104472

**Published:** 2023-02-16

**Authors:** Kuan Rong Chan, Clara W.T. Koh, Dorothy H.L. Ng, Shijie Qin, Justin S.G. Ooi, Eugenia Z. Ong, Summer L.X. Zhang, Huizhen Sam, Shirin Kalimuddin, Jenny G.H. Low, Eng Eong Ooi

**Affiliations:** a Program in Emerging Infectious Diseases, Duke-NUS Medical School, Singapore; b Department of Infectious Diseases, Singapore General Hospital, Singapore; c Key Laboratory of Pathogen Microbiology and Immunology, Institute of Microbiology, Chinese Academy of Sciences (CAS), Beijing, 100101, China; d Viral Research and Experimental Medicine Centre, SingHealth Duke-NUS Academic Medical Centre, Singapore; e Saw Swee Hock School of Public Health, National University of Singapore, Singapore

**Keywords:** COVID-19, MCEMP1, HLA-DRA, Transcriptomics, Biomarkers, Pathogenesis, Single cell sequencing, RNAseq, Gene expression, Systematic review

## Abstract

**Background:**

Mass vaccination has dramatically reduced the incidence of severe COVID-19, with most cases now presenting as self-limiting upper respiratory tract infections. However, those with co-morbidities, the elderly and immunocompromised, as well as the unvaccinated, remain disproportionately vulnerable to severe COVID-19 and its sequelae. Furthermore, as the effectiveness of vaccination wanes with time, immune escape SARS-CoV-2 variants could emerge to cause severe COVID-19. Reliable prognostic biomarkers for severe disease could be used as early indicator of re-emergence of severe COVID-19 as well as for triaging of patients for antiviral therapy.

**Methods:**

We performed a systematic review and re-analysis of 7 publicly available datasets, analysing a total of 140 severe and 181 mild COVID-19 patients, to determine the most consistent differentially regulated genes in peripheral blood of severe COVID-19 patients. In addition, we included an independent cohort where blood transcriptomics of COVID-19 patients were prospectively and longitudinally monitored previously, to track the time in which these gene expression changes occur before nadir of respiratory function. Single cell RNA-sequencing of peripheral blood mononuclear cells from publicly available datasets was then used to determine the immune cell subsets involved.

**Findings:**

The most consistent differentially regulated genes in peripheral blood of severe COVID-19 patients were MCEMP1, HLA-DRA and ETS1 across the 7 transcriptomics datasets. Moreover, we found significantly heightened MCEMP1 and reduced HLA-DRA expression as early as four days before the nadir of respiratory function, and the differential expression of MCEMP1 and HLA-DRA occurred predominantly in CD14+ cells. The online platform which we developed is publicly available at https://kuanrongchan-covid19-severity-app-t7l38g.streamlitapp.com/, for users to query gene expression differences between severe and mild COVID-19 patients in these datasets.

**Interpretation:**

Elevated MCEMP1 and reduced HLA-DRA gene expression in CD14+ cells during the early phase of disease are prognostic of severe COVID-19.

**Funding:**

K.R.C is funded by the 10.13039/501100001349National Medical Research Council (NMRC) of Singapore under the Open Fund Individual Research Grant (MOH-000610). E.E.O. is funded by the 10.13039/501100001349NMRC Senior Clinician-Scientist Award (MOH-000135-00). J.G.H.L. is funded by the 10.13039/501100001349NMRC under the Clinician-Scientist Award (NMRC/CSAINV/013/2016-01). S.K. is funded by the 10.13039/501100001349NMRC under the Transition Award. This study was sponsored in part by a generous gift from The Hour Glass.

**Research in context**

**Evidence before this study**

Worldwide vaccinations has significantly reduced the burden of severe COVID-19. However, waning immunity, especially in the elderly and immunocompromised, as well as emergence of immune-escape variants of concern, could lead to a re-emergence of severe COVID-19. However, it is unclear which prognostic biomarkers are most reliable in predicting the risk of severe disease progression.

**Added value of this study**

Through a systematic search and re-analysis of 7 publicly available transcriptomic datasets, this study uncovered that MCEMP1, HLA-DRA and ETS1 were most differentially regulated in severe COVID-19 patients as compared to mild COVID-19 patients. Of these genes, MCEMP1 and HLA-DRA were differentially expressed as early as four days before nadir of respiratory function. Single cell RNA sequencing revealed that the upregulation of MCEMP1 and downregulation of HLA-DRA transcripts in severe COVID-19 subjects were seen in CD14+ immune cell subsets, which are primarily myeloid lineage cells. This highlights that the early expansion of myeloid-derived suppressor cell gene signatures, particularly MCEMP1 and HLA-DRA, can be an early prognostic biomarker for COVID-19 disease severity.

**Implications of all the available evidence**

Our findings demonstrated that the induction of gene expression of MCEMP1 and downregulation of HLA-DRA in CD14+ cells can serve as early prognostic biomarkers for severe COVID-19.

## Introduction

The emergence of SARS-CoV-2 in year 2020 as a human respiratory pathogen resulted in a pandemic with high rates of morbidity and mortality globally. With mass vaccinations,^1^, ^2^, ^3^ the incidence of severe pulmonary disease has been dramatically reduced.^4^, ^5^, ^6^ However, cases of severe COVID-19 still remain, especially among the at-risk populations, such as the elderly and immunocompromised. Furthermore, immunity from both prior infection and vaccination has been shown to wane. Waning immunity, as well as the possible emergence of immune-escape variants of concern (VOC), could both result in the re-emergence of severe COVID-19 in the future. There thus remain an unmet need for reliable disease prognostic biomarkers to both triage COVID-19 cases at risk of disease progression at presentation, as well as for early detection of increasing trend of severe COVID-19 from either waning immunity or more pathogenic VOCs.

Severe COVID-19 is associated with impaired type-I interferon responses, followed by hyperinflammation that is characterised by heightened levels of proinflammatory cytokines and chemokines.^7^, ^8^, ^9^ This is accompanied by T-cell lymphopenia and suppression of T-cell functions^10^, ^11^, ^12^, ^13^ which can impair the host for clearing infected cells. Although these warning signs have improved clinical diagnosis of severe COVID-19, many of these clinical parameters often develop late during the course of disease and hence, their sensitivity and specificity as early biomarkers remain uncertain.^14^ Transcriptomic analyses have revealed widespread dysregulation of innate and adaptive immunity in severe COVID-19, including prominent neutrophil hyperactivation,^15^^,^^16^ production of monocytes with immunosuppressive characteristics^17^^,^^18^ and a marked decrease in T cells transcripts in the peripheral blood.^15^^,^^19^ However, these gene expression signatures have not been systematically analysed across multiple cohorts, and so it remains unknown which of these gene sets are more broadly generalisable and which of these transcripts are most suitable for early prognosis of severe COVID-19.

Herein, we performed a systematic review of publicly available transcriptomic datasets, with the goal of identifying early prognostic biomarkers that reliably differentiate severe and mild COVID-19 patients. We used a total of 7 publicly available RNAseq datasets, comprising of a total of 140 severe and 181 mild COVID-19 patients collected during the acute phase of SARS-CoV-2 infection, and uncovered that the top differential genes included MCEMP1, ETS-1 and HLA-DRA. In particular, MCEMP1 and HLA-DRA were found to be differentially expressed in severe COVID-19 patients as early as four days from nadir of respiratory function.^15^ Finally, from publicly available single-cell RNA sequencing datasets, we ascertained that the dysregulation of MCEMP1 and HLA-DRA transcripts occurs primarily in the CD14+ immune cell subsets. As upregulation of MCEMP1 and downregulation of HLA-DRA is characteristic of monocytic myeloid-derived suppressor (MDSCs),^17^ our systematic review support that the early expansion of MDSCs gene signatures, especially MCEMP1 and HLA-DRA, can be an early prognostic biomarker for COVID-19 disease severity.

## Methods

### Systematic search

Searches were conducted using the keywords “covid AND SARS AND transcriptomic∗ AND immune” on PubMed, Web of Science, Scopus, and Gene Expression Omnibus (GEO). Studies that performed whole-transcriptome analysis on whole blood or PMBCs in severe and mild acute COVID-19 patients were selected for further analysis. We excluded studies that use single-cell RNA analysis, review articles, studies which do not use PBMCs/whole blood for transcriptomic analyses and publications where datasets are not publicly available or in a re-analysable format. After filtering out these studies, we narrowed down to 8 clinical COVID-19 datasets where transcriptomics data were available for severe and mild COVID-19 subjects. The review protocol is registered in INPLASY (INPLASY2022110038) and is available in full on inplasy.com (https://doi.org/10.37766/inplasy2022.11.0038). The current study was carried out based on the guidelines and principles outlined by the PRISMA 2020 checklist. 1 researcher (D.N.H.L.) was involved in the systematic search process. Full details of the datasets are presented in Table 1. Risk of bias was assessed by 1 researcher (D.N.H.L.) in accordance with GRADE recommendations for observational studies^20^ (Supplementary Fig. 1).

**Table 1:** Summary of the individual datasets investigated.

Dataset	Sample type	Number of mild COVID-19 patients	Number of severe COVID-19 patients	Reference	Download resource
1	Whole blood	18	14	Fong et al., 2021	GSE155454
2	Whole blood	63	15	Bibert et al., 2021	https://doi.org/10.17632/8wxhhykfnh.2
3	Whole blood	10	6	McClain et al., 2021	GSE161731
4	Leukocytes	50	50	Overmyer et al., 2021	GSE157103
5	PBMCs	12	4	Arunachalam et al., 2020	GSE152418
6	Whole blood	23	46	Carapito et al., 2022	GSE172114
7	PBMCs	5	5	Zhang et al., 2021	GSE164805
8	Whole blood (Longitudinal)	4	6	Ong et al., 2021	E-MTAB-9721
9	PBMCs (Single cells)	4	6	Lee et al., 2020	GSE149689
10	BALF (Single cells)	3	6	Liao et al., 2020	GSE145926

Table contains the number of patients in the severe COVID-19 and mild COVID-19 subject groups, period of patient recruitment, and their respective database sources.

### Statistical analysis for transcriptomics data

Partek® Genomics Suite® 7.21.1119 was used to analyze the fold changes and p-values between the severe and mild COVID-19 patients in the different databases, to minimise random effects arising from different cohorts. Differentially expressed genes in the individual datasets were identified by filtering genes with fold change >1.3 and p-value <0.05. The top differentially regulated genes were defined by transcripts which were differentially expressed in at least 5 datasets. The raw counts of these genes were normalised by Z-score transformation, where combined scores were obtained by averaging Z-score values of MCEMP1 and HLA-DRA for individual patients.

### Building severe COVID-19 signature database with Streamlit

A database app was made using Streamlit (https://www.streamlit.io), which converts python codes into an interactive web tool. The online app is deployed at https://kuanrongchan-covid19-severity-app-t7l38g.streamlitapp.com/ and GitHub codes to build the web tool can be accessed at https://github.com/kuanrongchan/COVID19-severity.

### Longitudinal assessment of genes in COVID-19 patients

The time-course kinetics of gene expression across different time points spanning respiration nadir function was based on the study by Ong et al.^15^ The study also collected CRP and lymphocyte measurements from the patients, and we used these clinical parameters to correlate with MCEMP1 transcript abundance measured by microarray. Pearson correlation analyses were performed in Prism 9.2.0.

### Real-time PCR assessment of MCEMP1 in COVID-19 patients

Real-time PCR was performed using the samples obtained from the study by Ong et al.^15^ Complementary DNA synthesis was performed using qScript cDNA Synthesis Kit (Quantabio) and real-time PCR (Roche) was performed using MCEMP1 and B2M primers with the following sequences:

MCEMP1 Forward Primer: 5′-CTGAGATGTCCAAGGAGCTGCT-3′

MCEMP1 Reverse Primer: 5′-TGGTGATGCTCTGCTGAACGGA-3′

HLA-DRA Forward Primer: 5′-AGCTGTGGACAAAGCCAACCTG-3′

HLAD-DRA Reverse Primer: 5′-CTCTCAGTTCCACAGGGCTGTT-3′

B2M Forward Primer: 5′-CCACTGAAAAAGATGAGTATGCCT-3′

B2M Reverse Primer: 5′-CCAATCCAAATGCGGCATCTTCA-3′

The qPCR cycling conditions are as follows:

Denature: 95 °C

PCR (45 cycles): 95 °C for 10 s, 55 °C for 5 s, 72 °C for 10 s (single acquisition)

Melt curve: 95 °C for 5 s, 55 °C for 60 s, 97 °C (continuous acquisition)

Cooling: 40 °C for 10 s

### Single cell sequencing analysis datasets

Single-cell transcriptome data for COVID-19 in peripheral blood mononuclear cells (PBMC) (GSE149689) and bronchoalveolar lavage fluid (BALF) (GSE145926) were downloaded from the GEO database (https://www.ncbi.nlm.nih.gov/geo/).

### Single-cell data processing and analysis

Integrative analysis of single-cell data was performed using the Seurat R package (Version 3), and single-cell visualisation was performed using Uniform Manifold Approximation and Projection (UMAP). During quality control, cells with a mitochondrial gene ratio of more than 15% were removed, which may be potentially dead cells. Only those cells with gene numbers in the range of 200–5000 or RNA numbers detected between 1000 and 30,000 cells were retained. After quality control, 48,583 PBMCs and 78,666 BALF single cells were included in the subsequent analysis. Data were normalised using the Seurat package and a principal component analysis was performed taking the top 2000 genes with the largest coefficient of variation. The Harmony algorithm R package was used to remove batch effects between individual samples. After observing the cumulative error of different principal components (PCs) and the significance of each PC, the top 20 PCs were selected. The resolution parameter for cell clustering was set to 1. For analysis of the CD14+ subpopulations, similar analysis was performed, where the top 10 PC were used with clustering resolution of 0.5. The top differential genes were then evaluated by comparing MCEMP1+ and MCEMP1− cells.

### Effects of SARS-CoV-2 proteins on gene expression

Data was obtained from GSE186650. Epithelial cells were either transfected with SARS-CoV-2 proteins or GFP as a control. After 48 h, the transfectants were cocultured with monocytes and the gene expression changes were assessed at 14 h with RNAseq. Data was analysed by taking log2FC against the GFP controls. For conditions done in duplicates, the average value was obtained.

### Ethics approval and consent to participate

This clinical study was approved by the SingHealth Combined Institutional Review Board (2013/397/F) and the patients were enrolled for the study at Singapore General Hospital. All participants gave written informed consent, and study approval was obtained from the SingHealth Combined Institutional Review Board (CIRB 2017/2374).

### Statistics

Mean and 95% confidence interval (95% CI) statistics were calculated based on the data obtained from 7 transcriptomics datasets. Average log2FC values for severe vs mild COVID-19 were first calculated for each dataset. Thereafter, these values were used to plot forest plots using GraphPad Prism. For Pearson correlation analyses, normality was confirmed using the D'Agostino & Pearson test. The Pearson correlation coefficients and p-values were determined by GraphPad Prism (version 9.2.0). IFN scores were determined based on the expression of 28 type I IFN-stimulated genes, as described previously.^21^ Statistical comparisons between categorical variables were determined by unpaired t-test, and AUC measurements (confidence intervals, sensitivity, specificity, positive predictive values and negative predictive values) were determined by the GraphPad Prism (version 9.2.0) software. AUC values from the 7 transcriptomic datasets were used for plotting the mean and confidence intervals. Receiver-operating characteristic (ROC) curves were obtained through ROC analysis using GraphPad Prism (version 9.2.0) software. DeLong test was determined using the pROC R package.^22^ The Wilcoxon rank-sum test for single cell sequencing was evaluated in R. The meta R package was used to assess heterogeneity between study datasets.

### Data and code availability

All processed data can be readily downloaded from GitHub at https://github.com/kuanrongchan/COVID19-severity (https://doi.org/10.5281/zenodo.7114840), and accessed publicly at: https://kuanrongchan-covid19-severity-app-t7l38g.streamlitapp.com/.

### Role of funders

The funders have no role in study design, data collection, data analyses, interpretation or writing of report.

## Results

### MCEMP1, HLA-DRA and ETS1 gene expression levels were predictive of severe COVID-19 in publicly available transcriptomic datasets

We performed a systematic search for whole-genome expression datasets that examined whole blood or peripheral blood mononuclear cells (PBMCs) from patients with mild or severe acute SARS-CoV-2 infection (Supplementary Fig. S1). Up to 30 June 2022, our searches yielded 1015 potentially relevant studies. An additional 3 papers were identified via review of bibliographies, leading to a total of 1018 records. After exclusion of 415 duplicates and 534 studies after assessment of the titles and abstracts, we identified 66 studies for full text review. 61 studies were excluded after full text review because of the following reasons: review (n-11); not transcriptomic studies on PMBCs/whole blood in acute COVID-19 in humans (n = 21); did not compare mild vs severe acute COVID-19 (n = 15); datasets were not in a suitable format or were not publicly available (n = 7); or only contained single cell data (n = 7). We identified a total of 8 papers with transcriptomic analysis of whole blood or PMBC datasets comparing severe vs mild acute COVID-19.^9^^,^^15^^,^^23^, ^24^, ^25^, ^26^, ^27^, ^28^ Of these, 7 were included in for further analysis to identify signatures of severe vs mild COVID-19,^9^^,^^23^, ^24^, ^25^, ^26^, ^27^, ^28^ and one was used to study longitudinal gene expression kinetics.^15^ The patient demographics and time-points of blood sampling for the datasets are indicated in Supplementary Table S1.

**Fig. 1:**
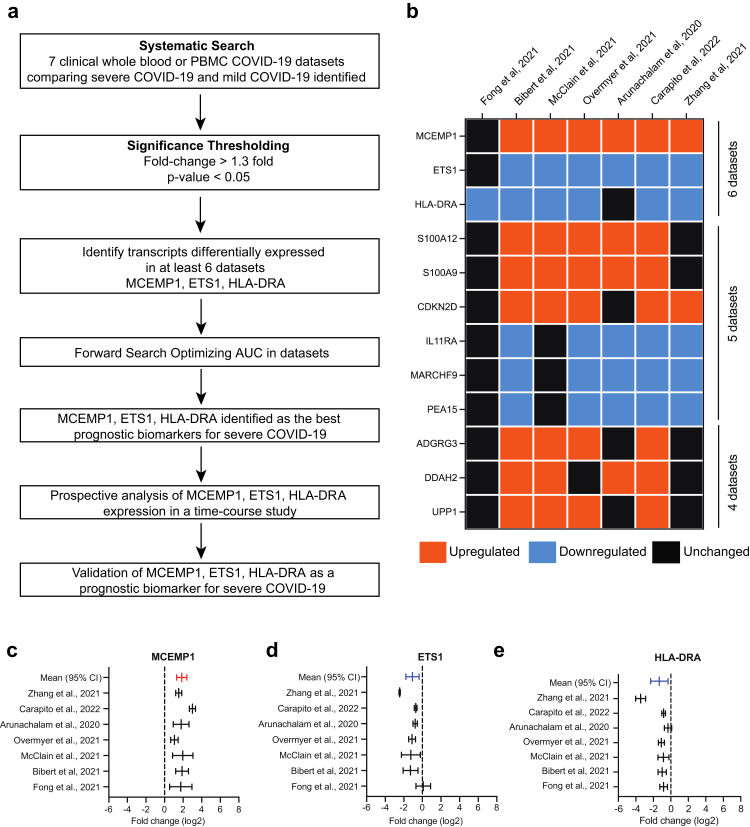
**Comparative analysis of transcriptomics datasets allows discovery of genes that are predictive of severe COVID-19. a,** Schematic of the multi-cohort analysis workflow, validation studies and single cell analysis that eventually identified MCEMP1 and HLA-DRA gene expression as the most consistent prognostic biomarker for severe COVID-19. **b,** Heatmap showing the number of datasets that the genes are differentially expressed (Fold-change > 1.3, p < 0.05) in severe compared to mild COVID-19 patients. Transcripts that were differentially regulated in 4 or more datasets are displayed. Orange indicates upregulated in the acute samples taken from severe compared to mild COVID-19 patients, blue indicates downregulated in severe compared to mild COVID-19 patients and black indicates no change. **c–e**, Forest plots of MCEMP-1, ETS1 and HLA-DRA transcript expression in severe COVID-19 patients compared to mild COVID-19 patients based on 7 transcriptomic datasets. x-axis represents the log2FC between severe and mild COVID-19 patients in the respective datasets. Whiskers represent the 95% CI. Mean statistics are obtained by first determining the average log2FC values for each dataset, followed by calculating the mean log2FC and 95% CI of these log2FC values. Red indicates upregulation in severe COVID-19 patients whereas blue indicates downregulation as compared to mild COVID-19 patients. Sample size for each dataset is indicated in Table 1.

Using our multi-cohort analysis framework (Fig. 1a), we evaluated the common genes which were differentially expressed in severe compared to mild acute COVID-19, based on fold change >1.3 and p < 0.05. The most consistent differentially upregulated gene in whole blood or PBMCs in 6 out of 7 datasets was MCEMP1, and the most consistently downregulated genes were ETS1 and HLA-DRA (Fig. 1b). Several other genes were identified to be significantly differentially expressed in 5 out of 7 datasets, such as upregulation of S100A12, S100A9 and CDKN2D, and downregulation of IL11RA, MARCHF9 and PEA15 (Fig. 1b). The biological description and function of the differentially expressed genes are shown in Supplementary Table S2. Among the top differentially regulated transcripts, MCEMP1 was upregulated by an average of 3.62-fold in severe COVID-19 patients compared to mild COVID-19 patients. On the other hand, ETS1 and HLA-DRA, were differentially downregulated by 2.10 fold and 2.28 fold, respectively, in severe COVID-19 patients (Fig. 1d and e). To allow users to conveniently search for individual gene expression differences between the severe and mild COVID-19 patients, we also developed a severe COVID-19 signature database (https://kuanrongchan-covid19-severity-app-t7l38g.streamlitapp.com/) based on our systematic review, where users can easily search for individual gene expression differences between severe and mild COVID-19 patients. The open-source software package is freely available at https://github.com/kuanrongchan/COVID19-severity, and users can modify these existing codes to add more databases or webtool features.

### MCEMP1 and HLA-DRA gene expression levels were differentially expressed in severe COVID-19 patients before nadir of respiratory function

**Fig. 2:**
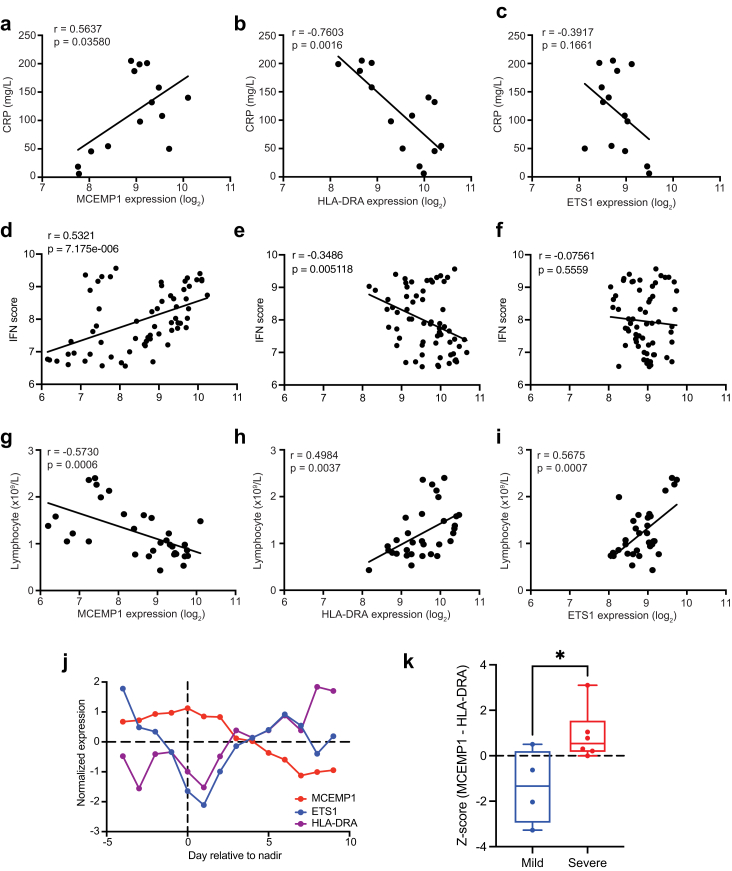
**MCEMP1 and HLA-DRA transcript expression levels can discriminate severe from mild COVID-19 patients as early as 4 days before nadir of respiratory function. a–f,** Scatterplots showing correlation between MCEMP1, HLA-DRA and ETS1 gene expression levels in whole blood that are associated with **a–c,** C-reactive protein (CRP), **d–f,** IFN score or **g–i**, lymphocyte counts. The respective Pearson's correlation (r) and p-values are shown. Patient samples are based on the study by Ong et al., 2021. **j,** Normalised expression levels of MCEMP1, HLA-DRA and ETS1 expression in severe and moderately severe COVID-19 patients (n = 6) over time relative to nadir of respiratory function (day 0), based on a study by Ong et al., 2021. **k,** Combination of MCEMP1 and HLA-DRA gene expression levels in mild COVID-19 (n = 4), and severe COVID-19 (n = 6) subjects as measured by real-time PCR. Combined scores are determined by subtracting individual Z-scores for MCEMP1 to Z-scores of HLA-DRA. Samples are taken on the first day of hospital admission for the severe and mild COVID-19 patients. Relative expression of each gene is normalised to B2M, which is the housekeeping gene. Normality was determined by the D′ Agostino & Pearson test (p > 0.05), and error bars indicate standard deviation of the data. ∗p < 0.05 (Unpaired t-test).

The comparative transcriptomics analysis indicated MCEMP1, ETS1 and HLA-DRA as the most promising candidates in discriminating severe vs mild COVID-19 outcomes. Since severe COVID-19 is characterised by increased C Reactive Protein (CRP) and lymphopenia,^29^, ^30^, ^31^, ^32^ which were used in several COVID-19 clinical severity scores, we correlated the abundance of MCEMP1, ETS1 and HLA-DRA transcripts to CRP and lymphocyte counts. For these measurements, we used patient samples from a recently published study that examined the temporal dynamics of these transcripts before and after the nadir of respiratory function.^15^ MCEMP1 transcript abundance was significantly correlated with CRP levels, while HLA-DRA was inversely correlated with CRP levels (Fig. 2a and b). No significant correlation was observed between ETS1 and CRP levels (Fig. 2c). Similarly, MCEMP1 and HLA-DRA, but not ETS1 expression, was significantly correlated with the type I IFN score, which captures the expression of 28 type I IFN-stimulated genes in myeloid and lymphoid cells^21^ and was previously demonstrated to be induced during severe COVID-19 and in children with multisystem inflammatory syndrome^33^ (Fig. 2d–f). Moreover, MCEMP1 was inversely correlated with lymphocyte counts, while ETS1 and HLA-DRA were positively correlated with lymphocyte counts (Fig. 2g–i), indicating the strong overall concordance of MCEMP1, ETS1 and HLA-DRA with the clinical predictors of severe disease outcome.

To assess the informativeness of MCEMP1, ETS1 and HLA-DRA throughout each phase of COVID-19, we examined the temporal dynamics of these transcripts before and after the nadir of respiratory function from the same study cohort. This nadir was defined as the day in which the patient was initiated on non-invasive or mechanical ventilation.^15^ Significantly heightened expression of MCEMP1 and downregulation of HLA-DRA expression were detectable 4 days before the nadir of respiratory function in patients who subsequently developed severe COVID-19 (Fig. 2j), supporting that differential expression of MCEMP1 and HLA-DRA is associated with severe disease outcome. To validate these findings, we performed qPCR on both MCEMP1 and HLA-DRA expression levels on first day of symptomatic onset. Indeed, the combination of both MCEMP1 and HLA-DRA expression levels was able to distinguish patients who subsequently developed severe vs mild COVID-19 (Fig. 2k). On the other hand, downregulation of ETS1 only occurred at the nadir of respiratory function (Fig. 2j). The transcript levels of these genes returned to baseline during recovery, approximately 7-8 days after the nadir of respiratory function (Fig. 2j).

### Assessing suitability of MCEMP1 and HLA-DRA gene expression levels in predicting severe COVID-19 and severe disease caused by other viruses

**Fig. 3:**
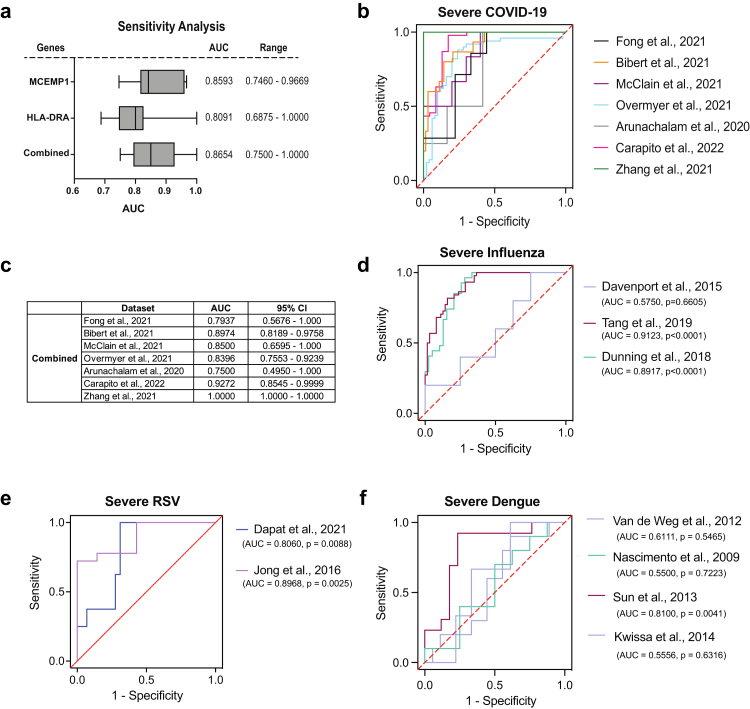
**Sensitivity of MCEMP1 and HLA-DRA across 7 transcriptomic datasets. a,** Box plots showing mean AUC and 95% CI of MCEMP1, HLA-DRA and combined scores (obtained by subtracting individual Z-scores for MCEMP1 to Z-scores of HLA-DRA) based on 7 severe vs mild COVID-19 transcriptomic datasets detailed in Table 1. AUC values were first obtained for each study, followed by calculation of the mean AUC and 95% CI of the AUC values. **b,** ROC curve showing suitability of using MCEMP1 and HLA-DRA combined scores in discriminating severe COVID-19 and mild COVID-19 patients in 7 datasets. **c,** Table showing AUC values and 95% CI for each dataset when both MCEMP1 and HLA-DRA expression levels were used for discriminating severe and mild COVID-19. **d–f,** ROC curve for comparing severe and mild **d,** influenza, **e,** RSV and **f,** dengue patients in 3 independent datasets based on combined scores of MCEMP1 and HLA-DRA expression levels. Sample size for the datasets are indicated in Table 1 and Supplementary Table S5.

To evaluate the suitability of MCEMP1 and HLA-DRA expression levels in discriminating severe COVID-19 from mild COVID-19 across multiple cohorts, we assessed the area under the curve (AUC) using combination of both MCEMP1 and HLA-DRA gene expression in the 7 independent transcriptomics study cohorts where blood was sampled immediately or at early time-points after hospital admission (Supplementary Table S1). MCEMP1 and HLA-DRA transcript levels displayed high AUC values (Mean AUC = 0.865 [95% CI 0.75–1.00]) (Fig. 3a), with the receiver-operator characteristic curve (ROC) plots showing that the combination of MCEMP1 and HLA-DRA was consistently able to distinguish severe from mild COVID-19 patients across all datasets (Fig. 3b and c, Supplementary Table S3). While the use of a combination of MCEMP1 and HLA-DRA did not significantly affect AUC values as compared to MCEMP1 and HLA-DRA alone, combination of both genes showed significantly higher AUCs than with HLA-DRA alone in the Carapito et al., 2022 dataset,^26^ suggesting that the combination of both of MCEMP1 and HLA-DRA could be more suitable for severe COVID-19 prognosis (Supplementary Fig. S2, Supplementary Table S4). We next explored if MCEMP1 and HLA-DRA expression levels could distinguish hospitalised patients with severe and mild influenza respiratory infection.^9^^,^^34^^,^^35^ MCEMP1 and HLA-DRA could discriminate severe from mild influenza respiratory infection although only in 2 out of 3 independent cohorts (Fig. 3d). Notably, MCEMP1 and HLA-DRA could also discriminate severe from mild disease caused by respiratory syncytial virus (RSV) in 2 independent studies^36^^,^^37^ (Fig. 3e), but not for dengue^38^, ^39^, ^40^, ^41^ (Fig. 3f). The datasets used for analyses were consolidated in Supplementary Table S5. Taken together, these findings highlight that MCEMP1 and HLA-DRA could be useful early prognostic biomarker for severe COVID-19 but likely limited for prognosis of severe influenza and dengue.

### Differential expression of MCEMP1 and HLA-DRA in severe COVID-19 patients occurred in predominantly in CD14+ cells

**Fig. 4:**
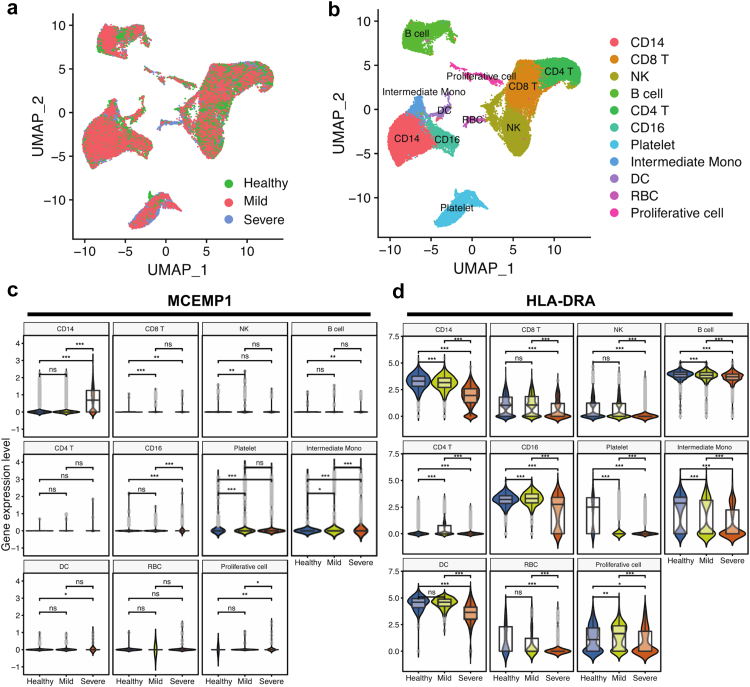
**Single-cell PBMCs transcriptomes highlight that differential expression of MCEMP1 and HLA-DRA occurs predominantly in CD14+ monocytes. a** and **b,** UMAP of 48,583 PBMCs based on GSE149689. Charts are coloured to show the **a,** disease severity status and **b,** cell type annotations. **c** and **d,** Violin plots showing the expression of **c,** MCEMP1 and **d,** HLA-DRA in different immune cell subsets in 4 healthy, 4 mild and 6 severe COVID-19 subjects. ∗p < 0.05, ∗∗p < 0.01, ∗∗∗p < 0.001 (Wilcoxon rank sum test).

To determine the immune cell types involved in the differential expression of MCEMP1, HLA-DRA and ETS1, we leveraged on a single cell transcriptome data of peripheral blood mononuclear cells (PBMCs) (GSE149689) that was performed on healthy controls (n = 4), and patients with mild COVID-19 (n = 4) and with severe COVID-19 (n = 6).^42^ We subjected 48,583 PBMCs to Uniform Manifold Approximation and Projection (UMAP) using the Seurat package^43^ and identified clusters that are assigned to 11 different immune cell types, including CD14+ classical and intermediate monocytes, CD16+ cells, dendritic cells (DC), CD4 and CD8 T cells, NK cells, B cells, platelets, red blood cells and proliferative cells in healthy, mild COVID-19 and severe COVID-19 patients (Fig. 4a and b). Comparing the MCEMP1 and HLA-DRA expression between the mild and severe cases in the different immune cell subsets revealed that high expression of MCEMP1 and low expression of HLA-DRA were seen pre-dominantly in CD14+ monocytes (Fig. 4c and d, Supplementary Fig. S3a). Similarly, CITE-seq data^44^ support that higher expression of MCEMP1 and reduced HLA-DRA was seen in conventional monocytes (Supplementary Fig. S3b). Moreover, high expression of S100A9 and S100A12, which were upregulated in 5 out of the 7 RNAseq datasets tested (Fig. 1b), was also seen in CD14+ monocytes (Supplementary Fig. S4), indicating an expansion of monocytes with immunosuppressive phenotype. Unlike MCEMP1, which was exclusively differentially expressed in CD14+ cells (Fig. 4c), reduced expression of HLA-DRA could be detected more broadly across other myeloid cells, B cells, T cells, NK cells and platelets, indicating a global suppression of antigen presentation in severe COVID-19 patients (Fig. 4d). Interestingly, lower ETS1 expression was only seen in proliferating cells but not the other immune cell subsets, which could potentially explain for the differences in temporal expression profiles as compared to MCEMP1 and HLA-DRA (Supplementary Fig. S5).

Given that MCEMP1 expression was dysregulated in CD14+ monocytes, we further examined if there were CD14+ subpopulations with higher transcript expression of MCEMP1. Based on the top 10 principal components, we identified 5 distinct CD14+ subpopulations (Supplementary Fig. S6a), where MCEMP1+ cells were detected in greater abundance in 3 of the 5 clusters (Clusters 0, 1, 3) (Supplementary Fig. S6b). Notably, MCEMP1 expressing cells also expressed higher transcript expression of S100 proteins (S100A8, S100A9, S100A12) with reduced IFITM1-3 expression and HLA-DRB1 (Supplementary Fig. S6c), further reinforcing that severe COVID-19 involves an early expansion of monocytes with an immunosuppressive phenotype.

To determine if the differential expression of MCEMP1 and HLA-DRA were seen in immune cells of the bronchoalveolar lavage fluid (BALF), we analysed 78,666 BALF single cells in 6 severe and 3 mild COVID-19 subjects based on a previously published dataset (GSE145926).^45^ 11 distinct clusters was detected, including macrophage, DC and plasmacytoid DC, CD4 and CD8 T cells, B cells, NK cells, mast cells and epithelial cells (Epi). While no differential expression of MCEMP1 and ETS1 was detected, reduced HLA-DRA expression was observed in macrophages, DC, mast cell, neutrophils, Epi and NK cells (Supplementary Fig. S7). Overall, our results indicate that reduced HLA-DRA can be detected in both peripheral blood and in BALF across multiple types of antigen-presenting cells, indicating that immunosuppression and reduced antigen-presentation could be critical early events involved in severe COVID-19 progression.

Finally, to shed insights into the potential mechanisms involved, we leveraged on a study conducted by Leon et al.,^46^ where epithelial cells were transfected with single SARS-CoV-2 proteins or with GFP as a control. After transfection for 48 h, transfectants were cocultured with monocytes and profiled by RNASeq. Interestingly, the majority of viral proteins caused upregulation of MCEMP1 and downregulation of HLA-DRA (Supplementary Fig. S8), indicating that soluble factors from infected cells could be responsible for the differential expression of these genes.

## Discussion

The host response to SARS-CoV-2, like many other acute viral infections, contributes to the pathogenesis of COVID-19. Some of these responses could thus serve as prognostic markers of severe COVID-19. Although the COVID-19 pandemic has now been controlled to a dramatic extent by mass vaccination, there remains populations at risk of severe pulmonary disease upon breakthrough infection. Furthermore, it is now also clear that vaccination cannot confer sterilising immunity. Consequently, SARS-CoV-2 will continue to evolve through human-to-human transmission and the risk of a VOC with heightened pathogenicity remains a concern in the coming years. A biomarker for early prognostication of severe COVID-19 is thus useful for case management and objective detection of increased rate of severe COVID-19 cases from new VOCs.

Several studies have detailed the molecular and cellular modifications associated with COVID-19 severity at the peak of their respiratory nadir, but few have studied the early time points of illness which could be informative in stratifying patients with increased risk of severe COVID-19 progression. Based on our systematic review and temporal characterisation of gene expression profiles, we ascertained that the induction of MCEMP1 and the downregulation of HLA-DRA gene expression was most reliable for early prediction of severe COVID-19. Our mined databases were collected from 5 countries (Singapore, Switzerland, USA, Hong Kong and France), indicating that these genes were unlikely to be influenced by underlying heterogeneity in the datasets such as age, genetic background, sample type (peripheral blood and PBMCs), and virus strains. Hence, peripheral blood MCEMP1 and HLA-DRA gene expression levels could be useful for early triaging of patients before severe COVID-19 disease progression.

Although the thrust of this work was to define early reliable prognostic biomarkers, we took advantage of the opportunity to analyse the publicly available single cell sequencing data from PBMCs and BALF to investigate the immune cell types involved. Interestingly, we found that the upregulation of MCEMP1 and downregulation of HLA-DRA were pre-dominantly detected in CD14^+^ monocytes, which is characteristic of monocytic myeloid-derived suppressor cells (MDSCs). In line with these observations, S100A9 and S100A12 which are known to be induced in MDSCs, were also upregulated in CD14^+^ monocytes of the severe COVID-19 subjects in the majority of the RNAseq datasets analysed.^47^ These gene signatures were also identified in the MS1 gene expression program, which was previously described as a MDSCs subset that were induced in patients with severe COVID-19.^17^ The increase in MDSCs levels could thus be a critical event that mediates the overall suppression of T cell responses that consequently exacerbates disease pathogenesis.^17^^,^^18^^,^^47^^,^^48^

Mast cell expressed membrane protein 1 (MCEMP1) is a transmembrane protein that is primarily expressed by mast cells and monocytes,^49^ and was characterised as a peripheral blood prognostic biomarker for patients with cerebral ischaemic stroke and cancer.^50^, ^51^, ^52^ Upregulation of MCEMP1 has also been shown to increase serum levels of proinflammatory cytokines such as TNF-α, IL-1β and IL-6 and increased T cell apoptosis, whereas decreasing the expression of MCEMP1 reduced inflammation in septic mice,^53^ which is in agreement with the hyperinflammatory responses observed in patients with severe COVID-19. While more studies will be required to pinpoint the role of MCEMP1 in monocytes, the promoter region of MCEMP1 contains NF-kB and NF-AT binding motifs,^49^ which may suggest that early dysregulation of NF-kB and NF-AT activation might have caused the early upregulation of MCEMP1 expression and expansion of MDSCs. Indeed, viral proteins of SARS-CoV-2, especially ORF3a, M, ORF7a and N has been demonstrated to directly induce NF-kB activation.^54^ Interestingly, transfection of these viral proteins in epithelial cells also resulted in the release of soluble factors that induced MCEMP-1 expression.^46^ Future studies that investigate MCEMP1 gene regulation, function and protein expression in monocytes after SARS-CoV-2 infection could thus shed valuable insights into the early molecular events that lead to severe COVID-19. In addition, as we have observed that 2 of the 5 severe COVID-19 patients in our cohort had indications of cardiac involvement, where one had myocardial infarction and the other had myocarditis with cardiomyopathy, it may also be worthwhile to investigate whether cardiac involvement caused by SARS-CoV-2 infection is related to MCEMP1 expression.

The downregulation of class II major histocompatibility complex gene HLA-DRA is observed in multiple cell types and in both PBMCs and BALF of severe COVID-19 patients, including myeloid cells, B cells, T cells, NK cells and platelets. The low expression levels of HLA-DRA globally indicates an overall immunosuppressive profile in blood and lungs of severe COVID-19 cases at early time-points, which can subsequently lead to compromised antigen presentation and T cell suppression that in turn promotes cytokine storm and hyperinflammation. These results are in consistent with previous findings documenting that severe COVID-19 is associated with the appearance of dysfunctional HLA-DR^lo^ CD163^hi^ and HLA-DR^lo^ S100A^hi^ CD14^+^ inflammatory monocytes, which may elevate immature and dysfunctional neutrophil frequencies^48^ and CD4+ T cell leukopenia.^32^ Our results are also consistent with other studies demonstrating that HLA-DRA protein expression is significantly reduced in severe COVID-19 patients compared to mild COVID-19 patients.^55^, ^56^, ^57^, ^58^, ^59^

Our study has several limitations. The datasets included in our analysis did not directly evaluate the effect of different SARS-CoV-2 variants on the transcriptomic responses to severe COVID-19, although the differences in disease severity was caused by different SARS-CoV-2 strains in one of our datasets analysed.^25^ We also noted heterogeneity in the study datasets included in the systematic review (Supplementary Fig. S9), so future studies will be useful to understand the heterogeneity in patients that may influence MCEMP1 and HLA-DRA gene expression. Moreover, as there are currently no studies documenting gene expression profiles more than 4 days before nadir of respiratory function, we were unable to determine precisely when the dysregulation of MCEMP1 and HLA-DRA expression occurs. Future longitudinal studies that characterise the early transcriptomic events should provide more information on the kinetics of MCEMP1 and HLA-DRA gene and protein expression over the entire course of infection. Future prospective studies will also be needed to validate the sensitivity and specificity of using MCEMP1 and HLA-DRA expression levels in predicting severe COVID-19.

### Conclusion

In conclusion, through systematic review and re-analysis of data from a total of 140 severe and 181 severe COVID-19 patients from 7 independent RNAseq datasets, time-series analysis and single cell sequencing RNAseq datasets, we identified that early MCEMP1 and HLA-DRA peripheral blood expression to be highly associated with progression to severe COVID-19.

## Contributors

C.W.T.K. and S.Q. analysed the RNAseq data and produced the figures. K.R.C. and C.W.T.K. developed the Streamlit webtool for database query. D.H.L.N. performed the systematic review. K.R.C. and J.O.S.G. verified the RNAseq data analysis and the systematic review. E.Z.O., H.S., S.K. and J.G.H.L. provided the clinical data parameters and samples for qPCR analyses. S.L.X.Z. performed the qPCR experiments. K.R.C., C.W.T.K., D.H.L.N., J.G.H.L. and E.E.O. conceived the study, wrote the manuscript and reviewed the manuscript. All authors have read and approved the final version of the manuscript.

## Data sharing statement

The raw data from the transcriptomics datasets can be accessed publicly and the resources, and the accession numbers are shown in Table 1 and Supplementary Table S5. All processed data can be publicly downloaded from GitHub at https://github.com/kuanrongchan/COVID19-severity (https://doi.org/10.5281/zenodo.7114840), and accessed publicly at: https://kuanrongchan-covid19-severity-app-t7l38g.streamlitapp.com/.

## Code sharing

Codes are publicly available and can be downloaded from GitHub at https://github.com/kuanrongchan/COVID19-severity (https://doi.org/10.5281/zenodo.7114840).

## Declaration of interests

The authors declare no competing interests.
