# Supplementary material for: Early peripheral blood MCEMP1 and HLA-DRA expression predicts COVID-19 prognosis
Source: eBioMedicine. 2023 Feb 16;89:104472. doi: 10.1016/j.ebiom.2023.104472 (PMC9934388; doi:10.1016/j.ebiom.2023.104472)
Supplement: Supplementary Tables and Figures [file mmc1.docx]

**Supplementary Fig. 1**

**Identification of studies**

Searched keywords “covid AND SARS AND transcriptomic* AND immune” on 30 June 2022

Records removed *before screening*:

Duplicate records (n = 415)

Records identified from*:

Databases:

PubMed (n=225)

Web of Science (n=242)

Scopus (n = 548)

Bibliography review (n = 3)

(n=1018)

**Identification**

Reports excluded:

Review (n=67)

Not primary immune response related (n=144)

Not transcriptomics studies on PMBCs/whole blood in acute COVID-19 in humans (n=283)

Only single cell data (n=26)

Preprint (n=14)

(n=534)

Records screened (titles and abstracts)

(n = 603)

**Screening**

Reports excluded:

Review (n=11)

Not transcriptomics studies on PMBCs/whole blood in acute COVID-19 in humans (n=21)

Did not compare mild vs severe acute COVID-19 (n=15)

Dataset not in suitable format or publicly available (n=7)

Only single cell data (n = 7)

(n=61)

Reports retrieved for full-text review

(n = 69)

Studies included in review

(n = 8)

**Included**

**Supplementary Fig. 2**

**Supplementary Fig. 3**

**Supplementary Fig. 4**

**Supplementary Fig. 5**

**Supplementary Fig. 6**

**Supplementary Fig. 7**

**Supplementary Fig. 8**

**Supplementary Fig. 9**

**Supplementary Table 1: Patient attributes for the different datasets from meta-analysis**

| **Dataset** | **Reference** | **Time of sampling** | **Age (Mean or Median)** | **BMI** | **Sex, male (%)** | **Co-morbidities (%)** | |
| --- | --- | --- | --- | --- | --- | --- | --- |
|  |  |  |  |  |  | **Diabetes** | **Hypertension** |
| 1 | Fong et al., 2021 | Admission | 48 (IQR: 32-64) | N.A. | 42.90 | 21.40 | 42.90 |
| 2 | Bibert et al., 2021 | <7 days after admission | 64 (Range: 23-82) | 30 (Range: 22-48) | 53.00 | 7.00 | 47.00 |
| 3 | McClain et al., 2021 | <10 days after admission | 46 (Range 20-91) | N.A. | 66.70 | 33.30 | 58.30 |
| 4 | Overmyer et al., 2021 | Admission | 62.9 (IQR: 55.0-73.0) | 30.92 (IQR: 24.5-32.05) | 66.70 | N.A. | N.A. |
| 5 | Arunachalam et al., 2020 | <14 days after admission | 55 (Range: 18-80) | N.A. | 58.00 | N.A. | N.A. |
| 6 | Carapito et al., 2022 | Admission | 41 (IQR: 34-46) | 30.2 (IQR: 27.1-35.6) | 76.00 | N.A. | N.A. |
| 7 | Zhang et al., 2021 | <9 days after admission | 51 (IQR: 19-74) | N.A. | 65.00 | 11.10 | 48.10 |

**Supplementary Table 2: Description and localisation of transcripts overexpressed in severe COVID-19 patients**

| **Gene** | **Name** | **Description** | **Tissue Expression Cluster (Human Protein Atlas)** |
| --- | --- | --- | --- |
| MCEMP1 | Mast cell expressed membrane protein 1 | Role in regulating mast cell differentiation or immune responses | Neutrophils - Inflammatory response |
| S100A12 | S100 calcium binding protein A12 | RAGE-binding protein involved in the regulation of inflammatory processes and immune response | Neutrophils - Humoral immune response |
| S100A9 | S100 calcium binding protein A9 | Calcium- and zinc-binding protein involved in the regulation of inflammatory processes and immune response | Bone marrow - Differentiation |
| ETS1 | ETS proto-oncogene 1, transcription factor | Directly controls the expression of cytokine and chemokine genes | T-cells - Adaptive immune response |
| HLA-DRA | Major histocompatibility complex, class II, DR alpha | Displays antigenic peptides on professional antigen presenting cells (APCs) for recognition by alpha-beta T cell receptor (TCR) on HLA-DR-restricted CD4-positive T cells | Macrophages - Immune response |
| ADGRG3 | Adhesion G protein-coupled receptor G3 | Regulates migration of lymphatic endothelial cells in vitro via the small GTPases RhoA and CDC42 | Neutrophils - Inflammatory response |
| CDKN2D | Cyclin dependent kinase inhibitor 2D | Interacts strongly with CDK4 and CDK6 and inhibits them | Bone marrow & Brain - Smell perception & Nucleosome |
| DDAH2 | Dimethylarginine dimethylaminohydrolase 2 | Role in the regulation of nitric oxide generation | Fibroblasts - ECM organization |
| KIFC3 | Kinesin family member C3 | Minus-end microtubule-dependent motor protein | Testis - Unknown function |
| UPP1 | Uridine phosphorylase 1 | Catalyzes the reversible phosphorylytic cleavage of uridine and deoxyuridine to uracil and ribose- or deoxyribose-1- phosphate | Esophagus - Epithelial junctions |
| IL11RA | Interleukin 11 receptor subunit alpha | Receptor for interleukin-11, where IL11/IL11RA/IL6ST complex may be involved in the control of proliferation and/or differentiation of skeletogenic progenitor or other mesenchymal cells | Skin - Epidermis development |
| MARCHF9 | Membrane associated ring-CH-type finger 9 | E3 ubiquitin-protein ligase that may mediate ubiquitination of MHC-I, CD4 and ICAM1, and promote their subsequent endocytosis and sorting to lysosomes via multivesicular bodies | Retina - Visual perception |
| PEA15 | Proliferation and apoptosis adaptor protein 15 | Blocks Ras-mediated inhibition of integrin activation and modulates the ERK MAP kinase cascade | Brain - Neuropeptide signaling |

**Supplementary Table 3: Sensitivity, specificity, positive predictive value and negative predictive value of MCEMP1 and HLA-DRA for severe COVID-19 prognosis**

| **Dataset** | **Reference** | **Sensitivity** | **Sensitivity (95% CI)** | **Specificity** | **Specificity (95% CI)** | **Positive Predictive Value** | **Negative Predictive Value** |
| --- | --- | --- | --- | --- | --- | --- | --- |
| 1 | Fong et al., 2021 | 1.00 | 0.65 – 1.00 | 0.56 | 0.27 – 0.81 | 0.64 | 1.00 |
| 2 | Bibert et al., 2021 | 0.87 | 0.62 – 0.98 | 0.79 | 0.68 – 0.88 | 0.50 | 0.96 |
| 3 | McClain et al., 2021 | 1.00 | 0.61 – 1.00 | 0.60 | 0.31 – 0.83 | 0.60 | 1.00 |
| 4 | Overmyer et al., 2021 | 0.88 | 0.76 – 0.94 | 0.76 | 0.63 – 0.86 | 0.79 | 0.86 |
| 5 | Arunachalam et al., 2020 | 1.000 | 0.51 – 1.00 | 0.58 | 0.32 – 0.81 | 0.44 | 1.00 |
| 6 | Carapito et al., 2022 | 0.98 | 0.89 – 1.00 | 0.83 | 0.63 – 0.93 | 0.92 | 0.95 |
| 7 | Zhang et al., 2021 | 1.00 | 0.57 – 1.00 | 1.00 | 0.57 – 1.00 | 1.00 | 1.00 |

**Supplementary Table 4. DeLong test to evaluate AUC differences between using HLA-DRA and MCEMP1 in combination, or individually**

| **Dataset** | **Reference** | **Gene tested** | **Z Statistic** | **p-value** |
| --- | --- | --- | --- | --- |
| 1 | Fong et al., 2021 | HLA-DRA vs Combined | -0.8216 | 0.4113 |
|  |  | MCEMP1 vs Combined | -0.2663 | 0.7900 |
| 2 | Bibert et al., 2021 | HLA-DRA vs Combined | -1.6583 | 0.0973 |
|  |  | MCEMP1 vs Combined | -1.1372 | 0.2554 |
| 3 | McClain et al., 2021 | HLA-DRA vs Combined | -0.9054 | 0.3652 |
|  |  | MCEMP1 vs Combined | 0.0000 | 1.0000 |
| 4 | Overmyer et al., 2021 | HLA-DRA vs Combined | -0.5577 | 0.5770 |
|  |  | MCEMP1 vs Combined | -0.8353 | 0.4036 |
| 5 | Arunachalam et al., 2020 | HLA-DRA vs Combined | -0.8864 | 0.3754 |
|  |  | MCEMP1 vs Combined | 0.9574 | 0.3384 |
| 6 | Carapito et al., 2022 | HLA-DRA vs Combined | -3.4724 | **0.0005** |
|  |  | MCEMP1 vs Combined | 1.2758 | 0.2020 |
| 7 | Zhang et al., 2021 | HLA-DRA vs Combined | 0.000 | 1.0000 |
|  |  | MCEMP1 vs Combined | -0.7071 | 0.4795 |

**Supplementary Table 5. Datasets for comparing severe and mild patients infected with influenza, RSV and dengue virus**

**Datasets used for Influenza Virus**

| **Dataset** | **Sample type** | **Number of mild Influenza patients** | **Number of severe Influenza patients** | **Reference** | **Download resource** |
| --- | --- | --- | --- | --- | --- |
| 1 | Peripheral blood | 63 | 44 | Tang et al., 2019 | GSE101702 |
| 2 | Whole blood | 39 | 27 | Dunning et al., 2018 | GSE111368 |
| 3 | Whole blood | 8 | 5 | Davenport et al., 2015 | GSE61754 |

**Datasets used for RSV**

| **Dataset** | **Sample type** | **Number of mild RSV patients** | **Number of severe RSV patients** | **Reference** | **Download resource** |
| --- | --- | --- | --- | --- | --- |
| 1 | Whole blood | 12 | 18 | Jong et al., 2016 | E-MTAB-5195 |
| 2 | Whole blood | 29 | 8 | Dapat et al., 2021 | GSE155925 |

**Datasets used for Dengue Virus**

| **Dataset** | **Sample type** | **Number of mild RSV patients** | **Number of severe RSV patients** | **Reference** | **Download resource** |
| --- | --- | --- | --- | --- | --- |
| 1 | PBMCs | 17 | 13 | Sun et al., 2013 | GSE43777 |
| 2 | Whole blood | 18 | 10 | Kwissa et al., 2014 | GSE51808 |
| 3 | Whole blood | 18 | 3 | Van de Weg et al., 2012 | E-MTAB-3162 |
| 4 | PBMCs | 8 | 10 | Nascimento et al., 2009 | GSE18090 |
